# Unique Contributions of an Arginine Side Chain to Ligand Recognition in a Glutamate-gated Chloride Channel*[Fn FN2]

**DOI:** 10.1074/jbc.M116.772939

**Published:** 2017-01-17

**Authors:** Timothy Lynagh, Vitaly V. Komnatnyy, Stephan A. Pless

**Affiliations:** From the Center for Biopharmaceuticals, Department of Drug Design and Pharmacology, University of Copenhagen, 2100 H Copenhagen, Denmark

**Keywords:** biophysics, glutamate, ion channel, membrane protein, neurotransmitter, Unnatural amino acids, arginine, ligand recognition, channel activation

## Abstract

Glutamate recognition by neurotransmitter receptors often relies on Arg residues in the binding site, leading to the assumption that charge-charge interactions underlie ligand recognition. However, assessing the precise chemical contribution of Arg side chains to protein function and pharmacology has proven to be exceedingly difficult in such large and complex proteins. Using the *in vivo* nonsense suppression approach, we report the first successful incorporation of the isosteric, titratable Arg analog, canavanine, into a neurotransmitter receptor in a living cell, utilizing a glutamate-gated chloride channel from the nematode *Haemonchus contortus*. Our data unveil a surprisingly small contribution of charge at a conserved arginine side chain previously suggested to form a salt bridge with the ligand, glutamate. Instead, our data show that Arg contributes crucially to ligand sensitivity via a hydrogen bond network, where Arg interacts both with agonist and with a conserved Thr side chain within the receptor. Together, the data provide a new explanation for the reliance of neurotransmitter receptors on Arg side chains and highlight the exceptional capacity of unnatural amino acid incorporation for increasing our understanding of ligand recognition.

## Introduction

Neurotransmitter receptors are vital signaling proteins that are embedded in the cell membrane and trigger intracellular changes in response to extracellular chemical signals. The two classical receptor types are metabotropic, G-protein-coupled receptors (GPCRs) that act over seconds or minutes via intracellular second messengers (1), and ionotropic, ligand-gated ion channels (LGICs)^2^ that mediate ion flux across the membrane on the millisecond timescale (2). The rapid chemo-electric signaling of LGICs is perfectly suited to the nervous system, where activation of sodium channels and chloride channels mediates excitatory and inhibitory signals, respectively (2). The first step in the process of activation is the recognition of a specific ligand, which in the case of the animal nervous system is very often the neurotransmitter glutamate (3).

Glutamate binding to neurotransmitter receptors has been studied in great detail, reflected in X-ray structures of ligand-receptor complexes of both LGICs (4, 5) and GPCRs (6). Perhaps not surprisingly, each complex contains an Arg side chain in close proximity to at least one of the glutamate carboxylates, suggestive of ionic interactions between negatively charged carboxylate and positively charged guanidino groups. Reduced function upon Ala substitution confirms the importance of these Arg residues in glutamate recognition in each receptor subfamily (7–9), but despite this apparent functional evidence for a charge-charge interaction, replacing a large Arg side chain with a much smaller Ala side chain involves more physico-chemical changes than merely removing a positive charge. As such, the precise contribution of highly conserved Arg side chains in ligand recognition remains unknown.

Here, we have sought experimental evidence for charge-charge interactions in ligand recognition, focusing on the AVR-14B glutamate-gated chloride channel (GluCl (10)). GluCls are invertebrate-specific members of the pentameric ligand-gated ion channel (pLGIC or “Cys-loop receptor”) family, sharing significant homology with vertebrate GABA and acetylcholine receptors and constituting an important antiparasitic drug target (11). Despite the fact that a *Caenorhabditis elegans* GluCl was the first eukaryotic pLGIC to be visualized by X-ray crystallography (5), the molecular basis for neurotransmitter recognition in GluCls has received little experimental interrogation, as compared with vertebrate homologs. It has recently been shown, however, that in GluCls, glutamate recognition involves interactions between aromatic residues on the principal face of the binding site with the glutamate amine (7), as well as interactions between Arg residues in the binding site with the glutamate carboxylate groups (12), as illustrated in supplemental Fig. S1. We replaced Arg^76^ in the glutamate binding site with a titratable amino acid, providing us with the unprecedented opportunity to test glutamate sensitivity when an isosteric side chain is present but charged or uncharged. The results indicate only a small role of positive charge and unveil another unique property of Arg side chains that contributes to glutamate sensitivity, namely the ability to form hydrogen bonds both with the agonist and with vicinal receptor side chains.

## Results

**FIGURE 1.**
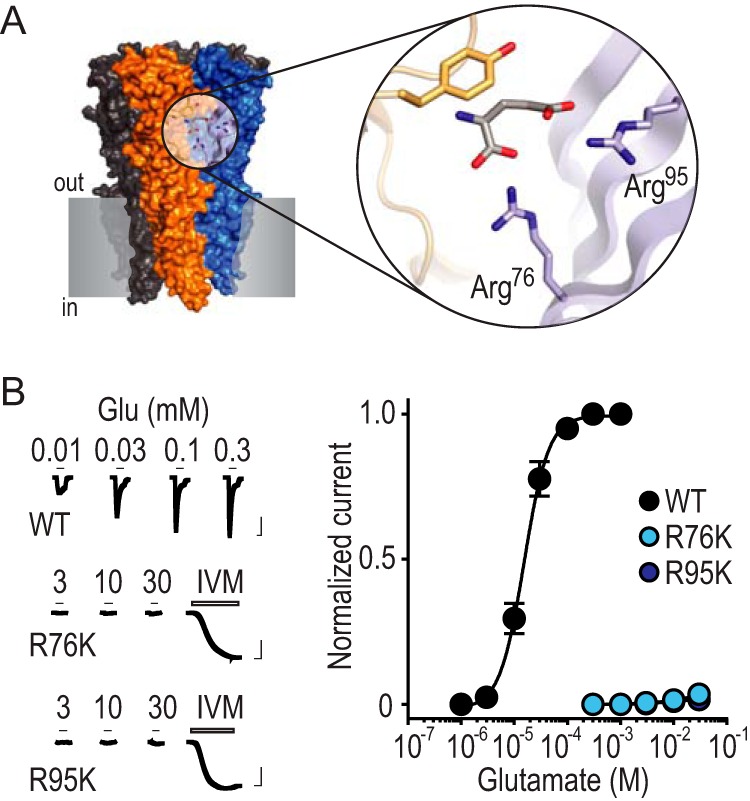
**Arg-Lys substitutions drastically reduce glutamate recognition.** *A*, X-ray structure of GLC-1 GluCl (PDB 3RIF; *gray shading*, notional cell membrane). Magnified view shows glutamate binding site and selected amino acid side chains. These include GLC-1 arginine residues 37 and 56, which are labeled Arg^76^ and Arg^95^ to describe the equivalent residues from the AVR-14B GluCl used in the present study. *B*, *left*, example recordings of glutamate (Glu) and ivermectin (IVM, 1 μm) responses at oocytes expressing mutant AVR-14B GluCls (*scale bars*: *x*, 5 s; *y*, 2 μA). Activation by IVM, which binds elsewhere on the receptor, confirms cell surface expression in the absence of Glu-gated currents. *Right*, mean ± S.E. (*n* = 4–8) peak current responses to increasing concentrations of Glu, normalized to maximum Glu-gated current (*WT*) or maximum IVM-gated current (mutants).

To test whether positive charge of Arg residues 76 and 95 is sufficient for glutamate recognition in the glutamate binding site of the AVR-14B GluCl, we replaced these individually with Lys via site-directed mutagenesis and measured glutamate-gated chloride currents with two electrode voltage clamp experiments (Fig. 1, *A* and *B*). Given the water-accessible location of these positions in GluCls (13) and the positive charge on Lys side chains in such environments (14), one would expect glutamate sensitivity of mutant receptors to reflect that of WT receptors if positive charge were the main contribution of these side chains. However, we observed that glutamate sensitivity was practically abolished at R76K and R95K mutants (Fig. 1*B*), suggesting that positive charge alone at these positions is not sufficient for high glutamate sensitivity.

**FIGURE 2.**
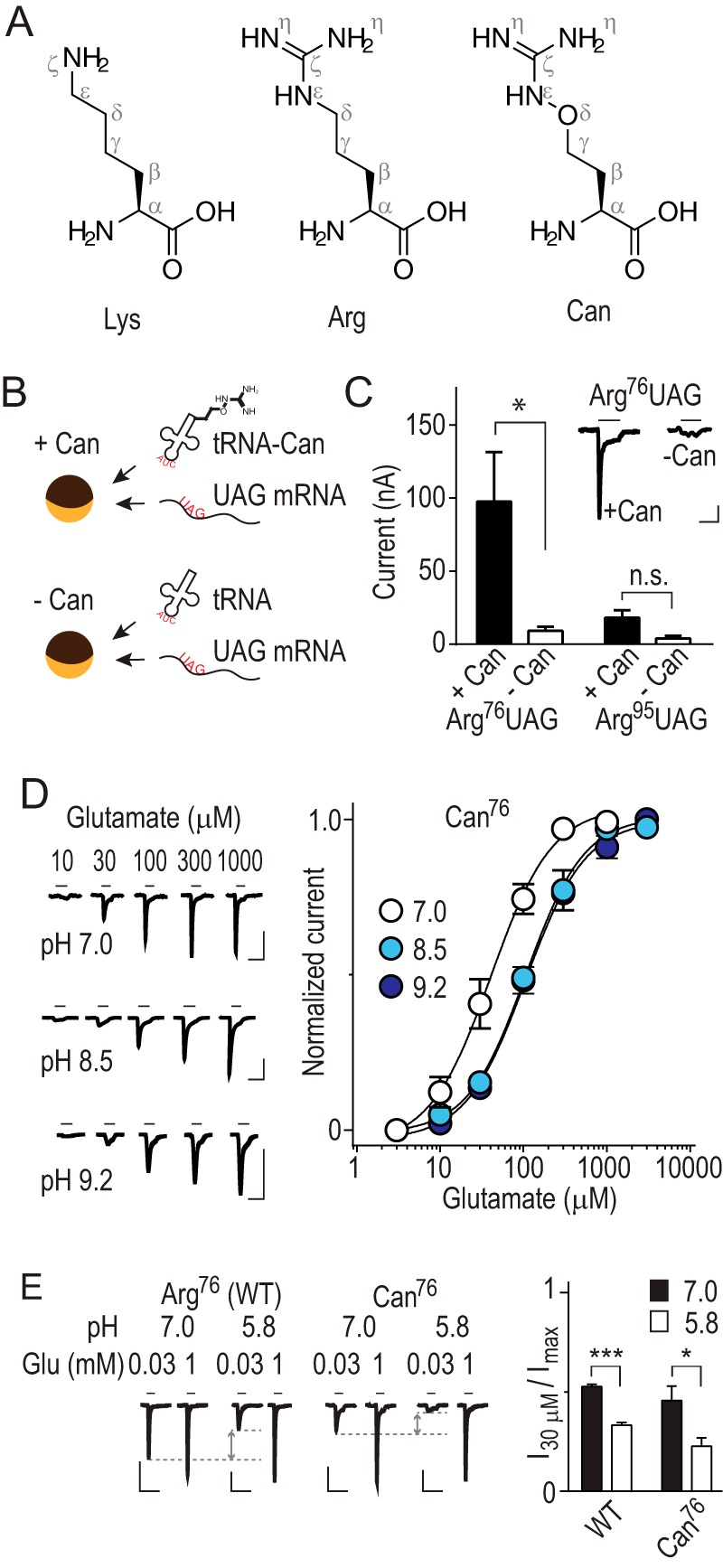
**Incorporation of titratable arginine analog, canavanine.** *A*, l-lysine (*Lys*), l-arginine (*Arg*), and l-canavanine (*Can*). *B*, graphic illustrating nonsense suppression of Arg^76^UAG mRNA by co-injection of Can-ligated tRNA into *Xenopus laevis* oocytes (*yellow/brown spheres*). *C*, mean peak current amplitude (± S.E.) in response to 10 mm glutamate at oocytes injected with mRNA and tRNA ± Can (*n* = 8–15; *, *p* < 0.05; *n.s.* not significant, ANOVA). *Inset* shows example responses to 10 mm glutamate at oocytes (pH = 7) injected with indicated RNA combinations (*scale bars*: *x*, 5 s; *y*, 20 nA). *D*, example recordings of current responses to increasing concentrations of glutamate and mean ± S.E. peak current responses (*n* = 5–8; normalized to maximum glutamate-gated current) at Can^76^ GluCls (Can^76^). *Scale bars*: *x*, 10 s; *y*, 100 nA. E, *left*, glutamate-gated currents at oocytes expressing wild-type or Can^76^ GluCls continuously perfused at pH 7.0 or 5.8, as indicated (*scale bars*: *x*, 30 s; *y*, 100 nA). *Right*, mean ± S.E. peak current responses to 30 μm glutamate, normalized to maximum glutamate-gated current (*I*_30 μm_/*I*_max_; *n* = 5–8; *, *p* < 0.05, ***, *p* < 0.001, Student's *t* test). *Gray arrows* illustrate inhibition of 30 μm glutamate-gated current. Can^76^ pH 7.0 recording in *E* is repeated from *D*.

Although Arg-to-Lys substitution largely retains side chain size and charge, it also involves the loss of two potentially hydrogen-bonding (H-bonding) nitrogen atoms (Fig. 2*A*), which could also underlie the loss of glutamate sensitivity we observed in R76K and R95K mutants. Assessing the precise contributions of charge or H-bonding of the Arg side chain to glutamate recognition would thus require substitution with an uncharged but otherwise isosteric analog. To this end, we sought to replace Arg^76^ or Arg^95^ with canavanine (Can), an isosteric Arg analog with a p*K_a_* of 7 (15) (Fig. 2*A*), reasoning that experiments at low and high pH would assay glutamate sensitivity when a single guanidino side chain is protonated or deprotonated, respectively. Site-specific incorporation of Can was achieved by the *in vivo* nonsense suppression method (16) (Fig. 2*B*). Successful incorporation of Can at position Arg^76^ was evident in robust glutamate-gated currents through Can^76^, receptors, but although currents were also observed for Can^95^, these were not significantly greater than controls lacking Can (Fig. 2*C*; 18 ± 5 nA, *n* = 10; 4 ± 2 nA, *n* = 8). This made further characterization of Can^95^ receptors difficult, and we chose not to investigate these further.

When we measured glutamate sensitivity of Can^76^ receptors at pH 7.0, at which one would expect only ∼50% of Can^76^ side chains to be protonated, we were surprised to find that the EC_50_ for activation by glutamate was 48 ± 7 μm (*n* = 6), and thus not significantly different from WT receptors (41 ± 11 μm, *n* = 8). At pH 8.5, when even fewer, if any, Can^76^ side chains are expected to be protonated, we observed only a modest decrease in glutamate sensitivity (Fig. 2*D*), with the EC_50_ significantly increased to 119 ± 11 μm (Table 1). A further increase in pH to 9.2 saw no additional rise in EC_50_ value (Fig. 2*D*; Table 1), suggesting that the effect was saturated around pH 8.5. No such pH-dependent shift was seen for WT receptors (Table 1), in which Arg^76^ residues are always protonated. This indicates that a 2-fold decrease in glutamate sensitivity in conditions that deprotonate the Can (not Arg) side chains is specific for receptors incorporating a Can residue in the glutamate binding site. Unfortunately, we could not measure the effects of fully protonated Can^76^ side chains, as acidic pH causes significant inhibition of function even in WT receptors (Fig. 2*E*), as is the case in structurally related GABA and glycine receptors (17, 18). To verify the 2-fold decrease in glutamate sensitivity observed with an uncharged analog at position 76, we attempted to replace Arg^76^ with citrulline, an analog in which one η nitrogen is replaced by an oxygen and which is uncharged (19), via nonsense suppression, but this was not successful (data not shown). Thus, and although a complete titration could not be completed, our data show that in conditions in which theoretically only 50% of Can^76^ side chains carry a positive charge, Can^76^ receptors show very similar glutamate sensitivity to WT Arg^76^ receptors (Table 1). Perhaps more strikingly, upon deprotonation and loss of positive charge in Can^76^ receptors, a mere 2-fold reduction in glutamate sensitivity is observed. This is a modest reduction in agonist sensitivity as compared with the 10,000-fold reduction caused by R76N or even R76K mutations in this very receptor (12) (Fig. 1), raising the possibility that a more substantial contribution to glutamate binding derives from some property of the Arg (or, indeed, Can) side chain other than positive charge.

**TABLE 1:** **Glutamate sensitivity of Can^76^ and Arg^76^ GluCls** For comparison with Can^76^ (oocytes injected with 40 ng of UAG mutant mRNA and tRNA-Can; Fig. 2), oocytes were injected with only 0.04 ng of Arg^76^ WT mRNA, to keep WT expression levels low, and I_max_ values comparable with Can^76^. ***, *p* < 0.001 as compared with pH 7.0 (ANOVA with Tukey's post test).

	EC_50_*^a^*	*I*_max_	*n*
	μ*m*	nA	
**Can^76^**			
pH 7.0	48 ± 7	60 ± 40	6
pH 8.5	119 ± 11***	214 ± 91	5
pH 9.2	118 ± 17***	110 ± 27	6
**Arg^76^ (WT)**			
pH 7.0	41 ± 11	241 ± 51	8
pH 8.5	43 ± 5	170 ± 47	8
pH 9.2	57 ± 7	428 ± 91	7

*^a^* EC_50_ value was calculated by plotting current amplitude against glutamate concentration and fitting with Hill equation for experiments at individual oocytes and then averaged. Data are mean ± S.E.

**FIGURE 3.**
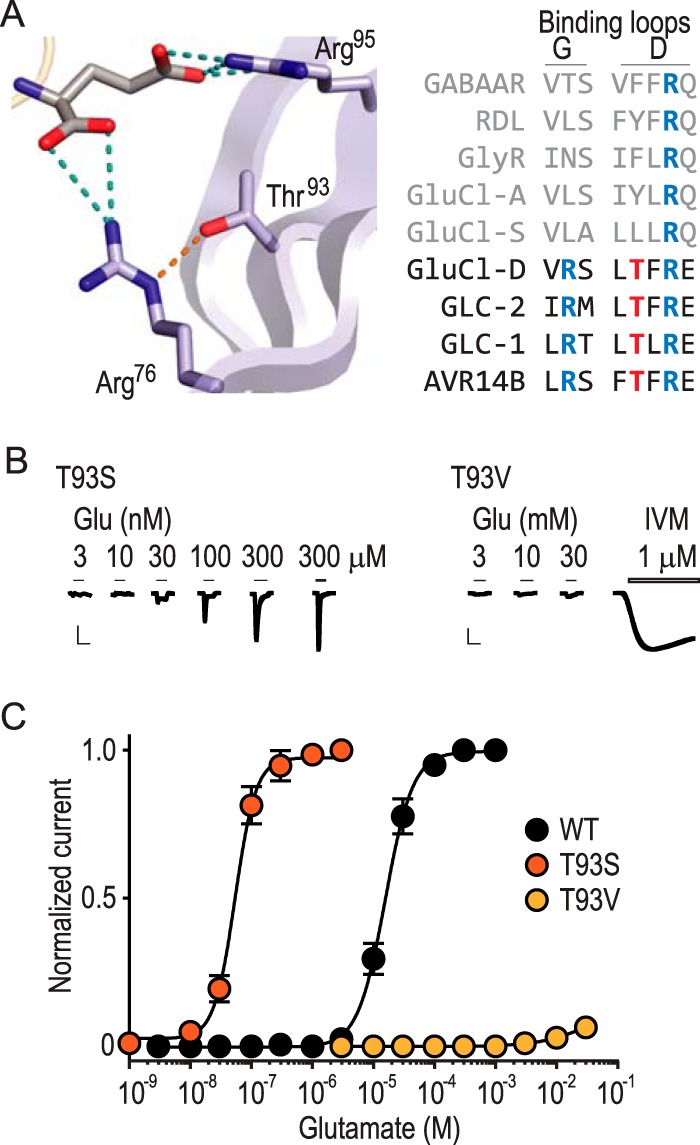
**Conventional mutagenesis shows the importance of Thr^93^ in glutamate recognition.** *A*, magnified view of GLC-1 crystal structure illustrating proximity of Thr^93^ hydroxyl oxygen to Arg^76^ ϵ nitrogen (2.9 Å; *orange dashed line*; *numbers* refer to equivalent residues in AVR-14B). *Dashed lines* indicate inter-atomic distances ≤3.5 Å. Amino acid sequence alignment shows selected Loop G and Loop D residues from ecdysozoan GluCls (*dark font*), lophotrochozoan GluCls and vertebrate and invertebrate GABA and glycine receptors (*light font*). *B*, example recordings of glutamate (*Glu*) and ivermectin (IVM) responses at oocytes expressing mutant AVR-14B GluCls (*scale bars*: *x*, 5 s; *y*, 2 μA). *C*, mean ± S.E. (*n* = 6–8) peak current responses to increasing concentrations of glutamate, normalized to maximum glutamate-gated current (WT and T93S) or maximum IVM-gated current (T93V).

Although some of that contribution is presumably via H-bonds between Arg (or Can) ηNH_2_ group(s) and glutamate (Fig. 3*A*), we considered that the Arg ϵNH group could also be important. Indeed, we noticed in the GLC-1 GluCl-glutamate X-ray structure (Protein Data Bank (PDB) 3RIF (5)) that although not in direct contact with the bound agonist, the hydroxyl oxygen atom of a Thr residue (equivalent to Thr^93^ in the AVR-14B GluCl) is located in close (2.9 Å) proximity to the ϵNH of the Arg equivalent to Arg^76^ (Fig. 3*A*). If this potential H-bond were important for glutamate sensitivity, we reasoned that the T93S substitution should retain glutamate sensitivity, as Ser also possesses a β-hydroxyl group. In contrast, Val is sterically similar to Thr but devoid of the hydroxyl, and mutant T93V receptors would be expected to show decreased glutamate sensitivity. Indeed, when we measured glutamate-gated currents at these mutants, T93V receptors showed drastically reduced glutamate sensitivity, barely responding to millimolar concentrations (Fig. 3, *B* and *C*). By contrast, T93S receptors showed a 370-fold *increase* in glutamate sensitivity as compared with WT (Fig. 3, *B* and *C*; Table 2), confirming that the hydroxyl at this position is required for regular (or increased) glutamate sensitivity, likely through an interaction with Arg^76^.

**TABLE 2:** **Glutamate sensitivity of WT and conventional mutants** Oocytes were injected with 10 ng of WT or mutant mRNA. ***, *p* < 0.001 as compared with WT (ANOVA with Tukey's post test).

	EC_50_*^a^*	I_max_*^b^*	*n*
	μ*m*	μA	
WT	17 ± 3	5.0 ± 0.5	4
R76K	≫100 mm*^c^*	0.10 ± 0.02	8
T93S	0.046 ± 0.011***	4.8 ± 0.7	6
R95K	≫100 mm*^c^*	0.05 ± 0.01	7
R76K/T93S	≫100 mm*^c^*	0.44 ± 0.09	8
R95K/T93S	530 ± 140	1.3 ± 0.4	9

*^a^* EC_50_ value was calculated by plotting current amplitude against glutamate concentration and fitting with Hill equation for experiments at individual oocytes and then averaged. Data are mean ± S.E.

*^b^* Maximum glutamate-gated peak currents. Mean ± S.E.

*^c^* For certain mutants, saturation in the concentration-response relationship was not reached, up to 30 mm glutamate. EC_50_ values are therefore estimated to be well over 100 mm.

**FIGURE 4.**
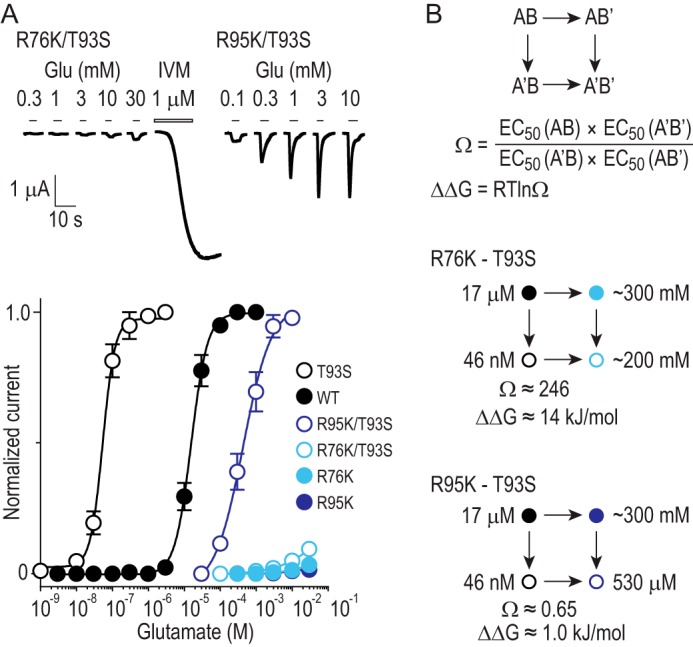
**Double mutant cycle analysis.** *A*, glutamate-gated currents at oocytes expressing double mutant T93S/R95K or R76K/T93S receptors and mean (± S.E.) data. Robust activation by IVM indicates successful expression of R76K/T93S despite small responses to glutamate. WT and single mutant data are repeated from earlier figures for comparison. *B*, principles of double mutant cycle analysis and analysis of Arg^76^–Thr^93^ and Arg^95^–Thr^93^ coupling. For two residues “A” and “B,” EC_50_ values of WT (AB), single mutant A′B, single mutant AB′, and double mutant A′B′ are used to calculate the coupling coefficient, Ω, from which the coupling energy, ΔΔ*G*, can be calculated (22). Our final values for Ω and ΔΔ*G* are only estimates because the EC_50_ values of certain single and double mutants could not be calculated: here the EC_50_ values have been estimated from *A*.

Seeking specific evidence for this interaction, we performed double mutant cycle analysis, according to which the T93S mutation should not restore high glutamate sensitivity on R76K mutant receptors because these WT residues are coupled and the effects of their mutation are not simply additive (20–22). In contrast, the combined effects of mutating independent residues are additive, and the T93S mutation might be expected to confer higher glutamate sensitivity on R95K receptors, as we do not expect coupling between these two residues. Indeed, double mutant T93S/R95K receptors responded to glutamate in a concentration range between T93S and R95K single mutants (Fig. 4*A*; Table 2), indicative of independence. R76K/T93S receptors, however, showed no greater glutamate sensitivity than single mutant R76K receptors (Fig. 4*A*), and analysis indicated an energetic coupling of ∼14 kJ/mol between Arg^76^ and Thr^93^ (Fig. 4*B*), which we interpret as evidence for a strong H-bond between the Arg ϵNH and Thr OH groups.

## Discussion

Taken together, these results suggest that the positive charge of Arg^76^ contributes little to glutamate binding in GluCls. Instead, our results show that two other aspects of the Arg side chain contribute to effective glutamate recognition. First, the data suggest that H-bonds between the Arg ηNH_2_ group(s) and the agonist α-carboxylate are important, as conventional Asn and Lys mutations that remove this moiety of Arg are severely detrimental to glutamate recognition, whereas the non-canonical substitution of Can, which retains ηNH_2_ groups, retains WT-like glutamate sensitivity. Second, the ϵNH of Arg appears to interact closely with a vicinal receptor hydroxyl side chain, the removal of which via Val substitution drastically reduces glutamate sensitivity. Based on available GluCl structures, this interaction is likely to stabilize Arg for its interaction(s) with the glutamate α-carboxylate (Fig. 3*A*). Notably, this Loop D hydroxyl side chain is highly conserved in GluCls that possess the *Loop G* Arg (Arg^76^ in AVR-14B), which interacts with the glutamate α-carboxylate. By contrast, the Loop D hydroxyl is absent in GluCls where instead a *Loop A* Arg (on the opposing face of the binding site, equivalent to Q141 in AVR-14B) interacts with the glutamate α-carboxylate (Fig. 3*A*) (12).

Conventional mutagenesis is an indispensable tool for dissection of protein structure and function, but in fine-tuning the details of ligand recognition, it is limited by the numerous physico-chemical changes involved in most substitutions (23). This is perhaps especially the case for Arg, where conventional analogs Lys and His are noticeably smaller and more frequently than Arg uncharged in physiological settings (14, 24). Previous attempts to circumvent the limitations of conventional mutagenesis by incorporating unnatural Arg analogs have been few (19, 25, 26) and arguably difficult (27). Our use of the *in vivo* nonsense suppression method was successful for one of the two positions tested, and despite limited efficiency (currents through Can^76^ were substantially smaller than conventional mutant receptors; compare *I*_max_ in Tables 1 and 2), the data strongly support the notion of robust and specific Can incorporation in three ways. First, the level of nonspecific incorporation of endogenous amino acids, as inferred from the very low current levels upon co-injection of uncharged tRNA, was very low (Fig. 2*C*). Second, although all conventional replacements at position 76 tested here and elsewhere (7, 12) resulted in drastic losses in glutamate sensitivity, we found Can incorporation to yield WT-like glutamate sensitivity (at pH 7.0; Table 1). Lastly, and perhaps most significantly, we found the agonist sensitivity of Can^76^ receptors to be titratable between pH 7.0 and 8.5, a property that would be highly unlikely in the event of endogenous amino acids incorporating into this position. We note here that in our hands the incorporation of Can was more successful than citrulline at positions 76 and 95. However, we cannot assess with certainty the reason for this difference, which could be due to ribosomal recognition of the charged tRNA, protein folding, or protein function.

In conclusion, Can incorporation, together with subsequent conventional mutagenesis, has unveiled crucial determinants of ligand recognition that had previously escaped identification. Our results suggest that the common occurrence of Arg residues in glutamate binding sites is related to the ability of Arg side chains to participate in H-bonds both with ligand and with vicinal receptor side chains simultaneously. Thus, the unique propensity of Arg for forming multiple H-bonds, as described previously in the context of intramolecular interactions in other protein families (28), seems to have been utilized by GluCls for the specific functional requirements of ligand recognition. The results also complement work on tetrameric ionotropic glutamate receptors (iGluRs), regarding both the unique role of Arg and the occurrence of vicinal hydroxyl side chains that could stabilize binding site architecture. In NMDA-type iGluRs, for example, Lys substitution of the Arg residue that binds the ligand α-carboxylate abolishes glutamate sensitivity (29, 30), much like R76K and R95K substitutions in the GluCl. Remarkably, NMDA-type iGluRs also contain Ser and Thr residues, whose side chain hydroxyl oxygens are, similar to Thr^93^ in the GluCl, as close as 2.7 Å to other side chains that form the glutamate binding site (29, 31), and whose substitution for Ala drastically reduces glutamate sensitivity (29).

We present here, to our knowledge, the first example of Arg analog incorporation into membrane-bound receptors, and as such, these results describe an incisive approach to dissecting chemical interactions in a broad and therapeutically relevant family of membrane proteins. Interestingly, an H-bond network adjacent to the ligand binding site has been proposed for the structurally related glycine receptor (32), suggesting that stabilization of ligand binding by such H-bond networks could be a conserved feature of ligand recognition by pLGICs.

## Experimental Procedures

### 

#### 

##### Chemical Synthesis

All reagents were of analytical grade and were used directly as received. The reagents were purchased from Sigma-Aldrich, unless stated otherwise. LC-MS analyses of synthesized compounds were performed on a Waters ACQUITY ultra high performance liquid chromatography system. Eluents A (0.1% HCOOH in water (v/v)) and B (0.1% HCOOH in acetonitrile (v/v)) were used in a linear gradient (100% A to 95% B) in a run time of 2.5 at a flow rate of 0.6 ml/min.

##### Nvoc-Can(Nvoc)-OH

Nvoc-Can(Nvoc)-OH was synthesized according to the procedure used elsewhere (33). l-Canavanine (l-α-amino-γ-(guanidinooxy)-*n*-butyric acid, 50 mg of H-Can-OH) was suspended in 1,4-dioxane (5 ml) along with sodium bicarbonate (120 mg, 5 eq). The resulting mixture was cooled down on ice bath, and NVOC-Cl (4,5-dimethoxy-2-nitrobenzyl chloroformate, 175 mg, 2,2 eq) was added in one portion. The reaction mixture was left stirring overnight. Water (5 ml) was added, the resulting mixture was extracted with ethyl acetate (3 × 10 ml), and the organic layer was discarded. The water layer was acidified with 1 m HCl to pH 4, and then the compound was extracted with ethyl acetate (3 × 20 ml), and the combined organic layer was dried over sodium sulfate and concentrated *in vacuo*. The resulting yellow oil (32 mg) was used directly in the next step. ESI MS calculated for C_25_H_31_N_6_O_15_ (M+H)^+^ 655.18; found 655.2.

##### Nvoc-Can(Nvoc)-OCH_2_CN

The compound from the previous step was dissolved in 1 ml of chloroacetonitrile, and triethylamine (145 μl, 1 mmol) was added. The reaction was stirred at room temperature overnight. Chloroacetonitrile was then removed *in vacuo*, and residual oil was dissolved in ethyl acetate (2 ml) and triturated with glass pipette. Precipitate of triethylamine hydrochloride was filtered off and washed with an additional portion of ethyl acetate (5 ml). Combined filtrate was concentrated under reduced pressure and dried overnight *in vacuo*. Obtained cyanomethyl ester (white to yellow solid, 36 mg) was used without further purification. ESI MS calculated for C_27_H_32_N_7_O_15_ (M+H)^+^ 694.20; found 694.1.

##### Nvoc-Can(Nvoc)-OpdCpA

pdCpA (5′-O-phosphoryl-2′-deoxycytidylyl-(3′→5′)adenosine (GE Healthcare/Dharmacon, 10 mg) was suspended in 200 μl of dry *N*,*N*-dimethylformamide, and Nvoc-Can(Nvoc)-OCH_2_CN (20 mg) and then NBu_4_OAc (10 mg) were added. The reaction was shaken at 37 °C until pdCpA was fully dissolved. Another portion (10 mg) of pdCpA was added, and the reaction was shaken at 37 °C until pdCpA was almost fully reacted (ultra performance liquid chromatography MS). 1 ml of ice-cold ether was added to the reaction mixture, and precipitate was collected by centrifugation. The residue was dissolved in 100 μl of acetonitrile and precipitated with additional 1 ml of ice-cold ether. White-to-yellow residue was dried in an N_2_ stream. The compound was purified by preparative reverse-phase HPLC on a C18 Phenomenex Luna column (250 × 20 mm, 5 μm, 100 Å) on an Agilent 1260 LC system equipped with a diode array UV detector and an evaporative light scattering detector. Eluents A (0.1% TFA in H_2_O (v/v)) and B (0.1% TFA in MeCN (v/v)) were used in a linear gradient (100% A to 100% B) in a run time of 20 min and with a flow rate of 20 ml/min. Fractions, containing UV signatures for both NVOC group and pdCpA, were collected, acetonitrile was removed *in vacuo*, and the resulting water solution was lyophilized. The obtained residue (2.6 mg) was dissolved in DMSO (100 μl), and the final concentration was adjusted to 3 mm by adding an additional volume of DMSO. ESI MS calculated for C44H51N14O26P2 (M-H_2_O+H)^+^ 1253.26, C44H50N14O26P2Na (M-H_2_O+Na)^+^ 1275.24, found 1253.3, 1275.3.

##### Expression of GluCls

All reagents were from Sigma-Aldrich, unless otherwise stated. The cDNA of the AVR-14B GluCl from *Haemonchus contortus* in the pT7TS vector (10) was used for site-directed mutagenesis with custom-designed primers (Eurofins Genomics) and PCR with PfuUltra II Fusion HS DNA Polymerase (Agilent Technologies). cDNA was linearized with XbaI (New England Biolabs), and mRNAs were synthesized with the Ambion mMESSAGE mMACHINE T7 transcription kit (Thermo Fisher Scientific) and purified in RNeasy columns (Qiagen). For incorporation of Can, we used the nonsense suppression method, where aminoacylated *Tetrahymena thermophila* tRNA (34) recognizing the amber stop codon UAG is co-injected into *Xenopus laevis* oocytes along with receptor mRNA containing a UAG mutation at the codon of interest (16), as follows. tRNA was prepared by ligation of full-length 5′ and 3′ DNA strands (Integrated DNA Technologies), RNA synthesis with T7-Scribe transcription kit (Cellscript), and purification with Chroma Spin DEPC-H_2_0 columns (Clontech). Aminoacylation of tRNA with Nvoc-Can(Nvoc)-OpdCpA was performed *in vitro* using T4 DNA ligase (New England Biolabs), and aminoacyl-tRNA was purified with phenol-chloroform extraction and ethanol precipitation, air-dried, and stored at −80 °C until use. Immediately before injection into oocytes, aminoacyl-tRNA was resuspended in 1 μl of water, and NVOC was removed by 50-s exposure to UV light (400-watt xenon lamp, Newport).

Oocytes from *Xenopus laevis* frogs (anesthetized in 0.3% tricaine, according to license 2014-15-0201-00031, approved by the Danish Veterinary and Food Administration) were separated with forceps and then treated with 0.5 mg/ml Type I collagenase (Worthington Biochemical) in OR2 (in mm, 2.5 NaCl, 2 KCl, 1 MgCl_2_, 5 HEPES, pH 7.4 with NaOH) with continuous shaking at 200 rpm and 37 °C. These were incubated in OR2 at 18 °C until injection of mRNA. For conventional mutants and WT mRNAs, 10 ng, in a volume of 40 nl (diluted in water), was injected using a Nanoliter 2010 injector (World Precision Instruments). For Can incorporation, 40 ng of UAG-mutant mRNA together with aminoacyl tRNA, in a volume of 40 nl, was injected. For lower expression of WT (Table 1), 0.04 ng mRNA was injected. Oocytes were incubated in Leibovitz's L-15 medium (Life Technologies) with 3 mm l-glutamine, 2.5 mg/ml gentamycin, 15 mm HEPES (pH 7.4 with NaOH) until experiments.

##### Electrophysiological Recordings and Data Analysis

One day after mRNA injection, oocytes were placed one at a time in a custom-built chamber (35), perfused with bath solution (in mm, 96 NaCl, 2 KCl, 1.8 BaCl_2_, and either 5 mm HEPES to pH 7.0, 7.4, 8.5, or 9.2 with NaOH/HCl or 5 mm MES to pH 5.8 with NaOH/HCl) for two-electrode voltage clamp experiments. l-Glutamate (dissolved in bath solution) was applied for ∼5 s with 1 min between subsequent applications using a ValveBank 8 perfusion system (AutoMate Scientific). Ivermectin was applied for longer until saturating current response was observed. Oocytes were clamped at −60 mV, and currents were recorded with microelectrodes filled with 3 m KCl, OC-725C amplifier (Warner Instruments), and Digidata 1550 digitizer (Molecular Devices) at 1 kHz with 200-Hz filtering. Peak current responses to l-glutamate were later analyzed in Clampfit 10 (Molecular Devices) with 10-Hz filtering for illustration. Peak current responses were plot against glutamate concentration using the four-parameter Hill equation in Prism 6 (GraphPad), and parameters were compared statistically (tests described in Table 1) using Prism 6.

##### Amino Acid Sequence Alignment

Amino acid sequences were retrieved from UniProt. Brief names (as in Fig. 3*A*), full names, and UniProt sequence IDs (in parentheses) are as follows: GABAAR, human GABA_A_R α1 subunit (P14867); RDL, *Drosophila melanogaster* RDL GABA receptor (P25123); GlyR, human glycine receptor α1 subunit (P23415); GluCl-A, *Aplysia californica* GluCl-2 (C7DLK0); GluCl-S, *Schistosoma mansoni* GluCl-2.1 (T2C5A6); GluCl-D, *D. melanogaster* GluCl (Q94900); GLC-2, *C. elegans* GluCl β (Q17328); GLC-1, *C. elegans* GluCl α (G5EBR3); and AVR14B, *H. contortus* AVR-14B GluCl (O46124). Sequences were aligned in MUSCLE (36) using default parameters in the European Bioinformatics Institute portal. For display in Fig. 3*A*, only three Loop G residues (AVR-14B/O46124 residues 75–77) and five Loop D residues (AVR-14B/O46124 residues 92–96) were shown.

## Author Contributions

All authors conceptualized the design and developed the methodology. T. L. and V. V. K. performed investigations. T. L. wrote the original draft, and all authors reviewed and edited the manuscript; T. L. performed visualization. T. L. and S. A. P. were responsible for funding acquisition; S. A. P. supervised the study.

## Supplementary Material

Supplemental Data

## Footnotes

2 The abbreviations used are:

LGIC: ligand-gated ion channel
pLGIC: pentameric ligand-gated ion channel
Can: canavanine
GluCl: glutamate-gated chloride channel
iGluR: ionotropic glutamate receptor
Nvoc: 4,5-dimethoxy-2-nitrobenzyl
pdCpA: 5′-O-phosphoryl-2′-deoxycytidylyl-(3′→5′)adenosine
ESI: electrospray ionization
IVM: ivermectin
Glu: glutamate
ANOVA: analysis of variance.
