# Supplementary material for: Unique Contributions of an Arginine Side Chain to Ligand Recognition in a Glutamate-gated Chloride Channel
Source: J Biol Chem. 2017 Jan 17;292(9):3940–6. doi: 10.1074/jbc.M116.772939 (PMC5339774; doi:10.1074/jbc.M116.772939)
Supplement: Supplemental Data [file supp_292_9_3940__index.html]

Unique contributions of an arginine side chain to ligand recognition in a glutamate-gated chloride channel — Unique Contributions of an Arginine Side Chain to Ligand Recognition in a Glutamate-gated Chloride Channel — Arginine Side Chain in Ligand Recognition — Supplemental Data 

# Unique Contributions of an Arginine Side Chain to Ligand Recognition in a Glutamate-gated Chloride Channel

## Supplemental Data

- Supplemental Figure S1 (.pdf, 405 KB) - Supplemental Figure S1
