# Supplementary material for: Unique Contributions of an Arginine Side Chain to Ligand Recognition in a Glutamate-gated Chloride Channel
Source: J Biol Chem. 2017 Jan 17;292(9):3940–6. doi: 10.1074/jbc.M116.772939 (PMC5339774; doi:10.1074/jbc.M116.772939)
Supplement: Supplemental Data [file 10.1074_M116.772939_jbc.M116.772939-1.pdf]

Unique contributions of an arginine side chain to ligand recognition in a glutamate-gated chloride channel

Timothy Lynagh, Vitaly V. Komnatnyy, Stephan A. Pless

Supplemental Data (one figure and references)

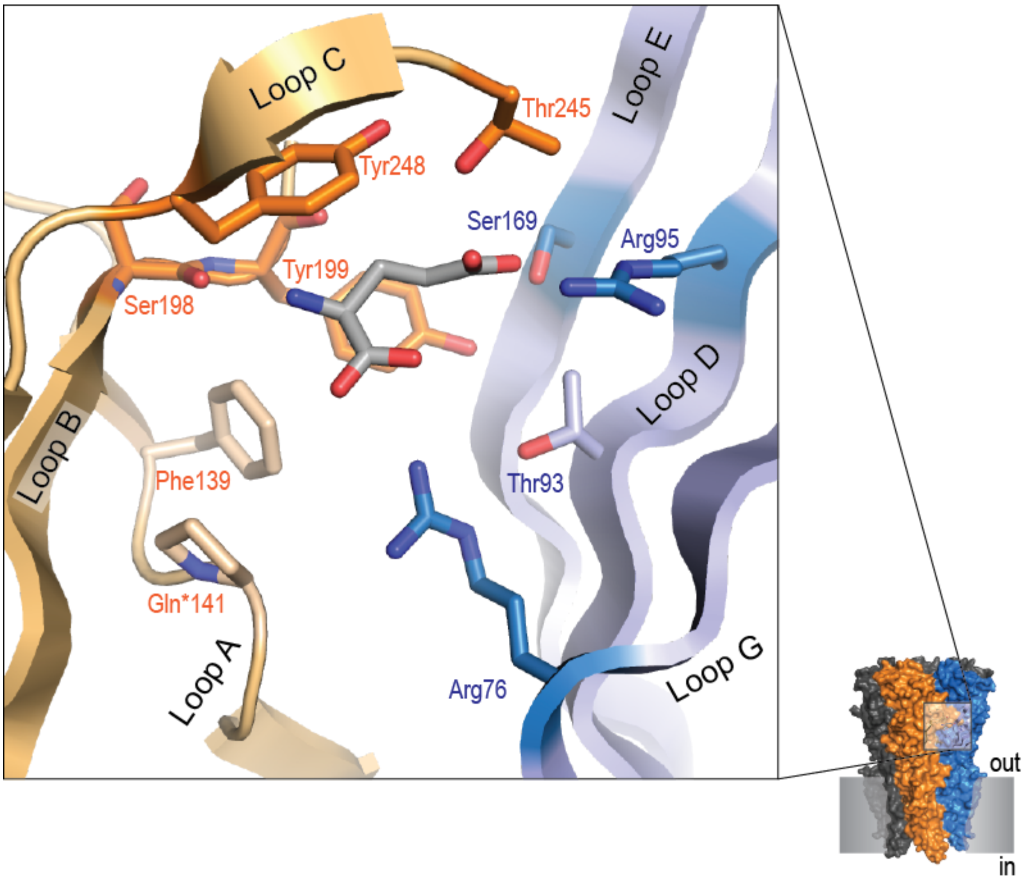

Supplemental Figure S1. Overview of determinants of glutamate recognition in glutamate-gated chloride channels (GluCls). Magnified view of glutamate (grey, middle) binding site in *Caenorhabditis elegans* GLC-1 GluCl X-ray structure (PDB 3RIF; (1)). Numbering refers to equivalent residues in the *Haemonchus contortus* AVR-14B GluCl used in the present study (asterisk indicates that GLC-1 Pro residue is equivalent to Gln141 in AVR-14B (2)). Principal face from one subunit in orange; complementary face from adjacent subunit in blue. Part of Loop C (principal face) and all of Loop F (complementary face) have been removed from the foreground for clarity. Highlighted side chains (darker orange and blue) and backbone atoms (Ser198 and Tyr199) are thought to contribute to glutamate sensitivity based on this crystal structure (1) and/or on mutagenesis experiments (2,3). Three other residues are shown, either to illustrate overall architecture (Phe139), to show the Loop A Gln referred in the *Discussion* (Gln141), or to illustrate the position of Thr93, referred to extensively in the main text.

Supplemental References

1. Hibbs, R. E., and Gouaux, E. (2011) Principles of activation and permeation in an anion-selective Cys-loop receptor. *Nature* **474**, 54-60
2. Lynagh, T., Beech, R. N., Lalande, M. J., Keller, K., Cromer, B. A., Wolstenholme, A. J., and Laube, B. (2015) Molecular basis for convergent evolution of glutamate recognition by pentameric ligand-gated ion channels. *Sci. Rep.* **5**, 8558
3. Daeffler, K. N., Lester, H. A., and Dougherty, D. A. (2014) Functional evaluation of key interactions evident in the structure of the eukaryotic Cys-loop receptor GluCl. *ACS Chem. Biol.* **9**, 2283-2290
